# Does patella resurfacing really matter? Pain and function in 972 patients after primary total knee arthroplasty: An observational study from the Norwegian Arthroplasty Register

**DOI:** 10.3109/17453671003587069

**Published:** 2010-03-31

**Authors:** Stein Håkon Låstad Lygre, Birgitte Espehaug, Leif Ivar Havelin, Stein Emil Vollset, Ove Furnes

**Affiliations:** ^1^The Norwegian Arthroplasty Register, Department of Orthopaedic Surgery, Haukeland University Hospital; ^2^Department of Public Health and Primary Health Care, University of Bergen; ^3^Department of Surgical Sciences, University of Bergen, BergenNorway

## Abstract

**Background and purpose:**

Resurfacing of the patella during primary total knee arthroplasty (TKA) is often recommended based on higher revision rates in non-resurfaced knees. As many of these revisions are insertions of a patella component due to pain, and since only patients with a non-resurfaced patella have the option of secondary resurfacing, we do not really know whether these patients have more pain and poorer function. The main purpose of the present paper was therefore to assess pain and function at least 2 years after surgery for unrevised primary non-resurfaced and resurfaced TKA, and secondary among prosthesis brands.

**Methods:**

Information needed to calculate subscales from the knee injury and osteoarthritis outcome score (KOOS) was collected in a questionnaire given to 972 osteoarthritis patients with intact primary TKAs that had been reported to the Norwegian Arthroplasty Register. Pain and satisfaction on visual analog scales and improvement in EQ-5D index score **Δ**EQ-5D) were also used as outcomes. Outcomes were measured on a scale from 0 to 100 units (worst to best). To estimate differences in mean scores, we used multiple linear regression with adjustment for possible confounders.

**Results:**

We did not observe any differences between resurfacing and non-resurfacing in any outcome, with estimated differences of ≤ 1.4 units and p-values of > 0.4. There was, however, a tendency of better results for the NexGen implant as compared to the reference brand AGC for symptoms (difference = 4.9, p = 0.05), pain (VAS) (difference = 8.3, p = 0.004), and satisfaction (VAS) (difference = 7.9, p = 0.02). However, none of these differences reached the stated level of minimal perceptible clinical difference.

**Interpretation:**

Resurfacing of the patella has no clinical effect on pain and function after TKA. Differences between the brands investigated were small and they were assumed to be of minor importance.

## Introduction

There is an ongoing discussion regarding whether resurfacing of the patella during primary total knee arthroplasty (TKA) should be recommended or not. This has led to several observational and randomized studies, and eventually to meta-analyses (Forster 2004, Nizard et al. 2005, Pakos et al. 2005, Parvizi et al. 2005). The meta-analyses have included studies in which the main outcomes were risk of reoperation, level of anterior knee pain, and other knee scores. None of these reviews found firm evidence regarding superiority of resurfaced or non-resurfaced prostheses. However, these studies still reported indications of better results for resurfaced prostheses, mainly because of a lower risk of reoperation for resurfaced implants. A critical appraisal of available evidence found methodological limitations in all the studies examined and neither treatment option was clearly superior (Calvisi et al. 2009).

A previous observational study from the Norwegian Arthroplasty Register (NAR) found a 1.3 times higher but not statistically significantly elevated (p = 0.2) overall rate of revisions for non-resurfaced prostheses (Furnes et al. 2002). There was, however, a significantly (2.5-fold) higher rate of revision for infection in knees with resurfaced prostheses, while non-resurfaced prostheses had a 5.7-times higher risk of revision because of pain. Many of the revisions for pain involved addition of a patella component to the native patella. Since secondary resurfacing is an available option only in non-resurfaced knees, we do not really know whether there were any differences in perception of pain between the two treatment groups. Further investigation of patients' subjective pain and function would therefore be of value when assessing the quality of the two types of TKA.

The major goal of the present study was therefore to investigate whether the levels of function and pain are different for patella resurfaced and non-resurfaced, unrevised, total knee prostheses, and our secondary aim was to investigate whether function and pain vary with different prosthesis brands.

## Patients and methods

### The Norwegian Arthroplasty Register (NAR)

Practically all patients (99%) who receive a primary arthroplasty of the knee are reported to NAR (Espehaug et al. 2006). The register was established in 1987 as a hip prosthesis register, but from 1994 it was extended to cover all artificial joints including knee arthroplasty (Havelin et al. 2000, Furnes et al. 2002). NAR receives information directly from the orthopedic surgeons. Information on patient-related outcome such as pain and function is not reported to the register. To assess patients' perception of pain and function after undergoing TKA, we therefore invited selected individuals registered in the NAR to participate in a postal survey.

### Participants

**Figure 1.**
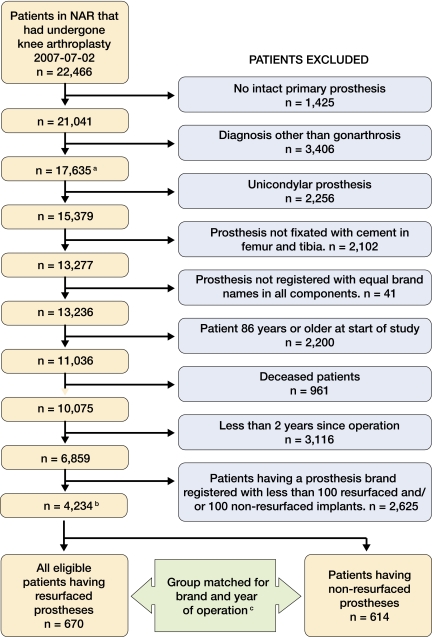
Description of the selection procedure.

Possible participants were patients registered in the NAR with at least 1 unrevised cemented primary TKA inserted due to gonarthrosis. The individuals should be aged 85 years or less, and the operation should have been performed at least 2 years prior to the survey to ensure that the result of the intervention had stabilized (Murray and Frost 1998, Burnett et al. 2004, Lingard et al. 2004). Only patients with a prosthesis brand already registered with at least 100 resurfaced and 100 non-resurfaced implants were eligible for inclusion. All patients with a resurfaced implant meeting these criteria were invited to participate in the study (134 with AGC, 186 with Genesis I, 238 with LCS, and 112 with NexGen). Since the use of non-resurfaced prostheses has increased and the use of resurfaced prostheses has decreased over the years in Norway, the selection of patients with non-resurfaced prostheses was matched according to brand and year of operation to ensure compatibility (134 AGC, 180 Genesis I, 238 LCS, and 62 NexGen). It was not possible to match all resurfaced NexGen prostheses with corresponding non-resurfaced prostheses since resurfaced prostheses were almost exclusively used early in the period. This led to similar numbers of patients with resurfaced (n = 670) and non-resurfaced (n = 614) prostheses, making a total of 1,284 individuals. A detailed account of the selection procedure is given in Figure 1.

**^a^** For patients with bilateral intact primary prostheses, only the most recent with gonarthrosis as diagnosis was eligible for inclusion. **^b^** Genesis I, AGC, LCS and NexGen were eligible for inclusion. **^c^** Resurfaced and non-resurfaced prostheses were matched for brand and year of operation. This led to exclusion of 2,950 patients with non-resurfaced implants.

After 2 months, a reminder was sent out to those who failed to respond to the initial questionnaire. In all, 972 patients completed the questionnaire, 305 either declined or did not respond, and 7 patients had died or were unable to be located by the post office.

### Questionnaire

The questionnaire consisted of the valid and reliable self-administrated instrument for calculation of the knee-specific knee injury and osteoarthritis outcome score (KOOS) (Roos and Lohmander 2003). A Norwegian translation from the Swedish version of KOOS was made for this study, and has been approved as the official Norwegian translation. A description of the validation process of this translation can be found at www.koos.nu.

To assess the potential effect of general health factors, the questionnaire also included questions needed to calculate the Charnley category applied to knee arthroplasty patients and the valid and reliable instrument for health quality measurement, the EQ-5D index score (Greiner et al. 2003). Information needed for calculation of preoperative and current EQ-5D index scores was given by the patients at the time when filling in the form. In addition, questions regarding patients' “satisfaction with the surgery”, and degree of “pain from the operated knee” were included. With the exception of the latter two, where a visual analog scale (VAS) was used, all questions had standardized answer options given as Likert boxes.

The study was approved by the Norwegian Data Inspectorate, Norwegian Social Science Data Services, and the Regional Committee for Research Ethics in Western Norway (date of issue: 02/23/2006, registration number: 046.06). The patients received the questionnaire together with an information letter, and returned the questionnaire to the register with a signed consent to participate in the study.

### Outcome measures

KOOS, which was used as primary outcome on patients' perception of pain and function, consists of 42 individual questions, making up 5 subscales: Pain, other symptoms (Symptoms), activities in daily living (ADL), function in sport and recreation (Sport&Rec) and knee-related quality of life (QOL). Only the previous week was to be considered when answering most of the questions, and each question received a score from 0 to 4. A normalized score (100 indicating no symptoms and 0 indicating extreme symptoms) was calculated for each subscale. Calculation of the scores and treatment of missing data were done in accordance with the description at www.koos.nu.

In addition, we used “pain from the operated knee” (Pain(VAS)) and “satisfaction with the operation” (Satisfaction (VAS)) as outcomes. In the analyses the VAS scores were reversed, with 100 indicating the best possible state and 0 indicating the worst possible state. Finally, improvement in quality of life (ΔEQ-5D), calculated as the difference between the present and preoperative EQ-5D index scores multiplied by 100, was used as outcome.

### Sensitivity analysis of potential bias due to different revision criteria from resurfaced and non-resurfaced implants

A bias may have been introduced if a non-resurfaced prosthesis was more likely to be revised than resurfaced (Australian Orthopaedic Association National Joint Replacement Registry. Annual Report. 2008, Furnes et al. 2002). This would have given a falsely low proportion of patients with poor results in that group. The potential effect of this was assessed in a sensitivity analysis where we included information also from patients with revised prostheses. This was possible due to the availability of information from another survey comprising all patients registered in the NAR with revised implants.

The original study material consisted of all intact resurfaced knees in the register that met the inclusion criteria, and about the same number of intact non-resurfaced knees matched on year of operation. To these we added all revised resurfaced knees that met the inclusion criteria (n = 23) and varying numbers of revised non-resurfaced implants. The latter were randomly selected from the 148 revised non-resurfaced knees that met the inclusion criteria. The Mann-Whitney test was used to compare pain and discomfort between the groups using the specific EQ-5D question regarding pain and discomfort (1 = best, 3 = worst). This information related to when the questionnaire was filled in for patients with intact prostheses, while patients with a revised prosthesis gave information in retrospect regarding their situation before the revision.

### Statistics

Minimal perceptible clinical difference is 8–10 units for KOOS subscales (Roos and Lohmander 2003) and 9–12 units on a visual analog scale (Ehrich et al. 2000). To have an 80% chance of detecting a significant (at the 2-sided 5% level) 10-point difference between the 2 groups in the mean KOOS subscales, with an assumed standard deviation of 20, 64 individuals in each treatment group were required. Thus, to ensure good representation for both treatment groups, a restriction on operation volume of each brand was set to at least 100 registered resurfaced and 100 registered non-resurfaced operations.

Differences in response rates were tested with the chi-squared-test. To estimate differences in mean outcome scores for non-resurfaced and resurfaced prostheses, we used multiple linear regression with adjustment for possible confounding by age (< 65, 65–70, 70–80, > 80), sex, preoperative EQ-5D index score (< 30, 0.30–0.69, > 0.69), Charnley category (A, B, C), and brand of prosthesis. We also investigated any differences within a particular prosthesis brand. Adjusted differences in mean values between resurfaced and non-resurfaced prostheses are presented with 95% confidence intervals (95% CIs) and p-values.

Multiple linear regression was also used to investigate any possible association between prosthesis brand and mean outcome scores. In these analyses, we also adjusted for time since operation. Adjusted differences in mean scores are presented with p-values relative to the AGC prosthesis brand. 9 patients who did not have the AGC Universal design were excluded from this analysis (5 resurfaced and 4 non-resurfaced).

In addition, all analyses were performed excluding prostheses with posterior cruciate ligament sacrificing design and also excluding constrained condylar prostheses (18 prostheses: 10 resurfaced and 8 non-resurfaced).

In the analyses, p-values less than 0.05 were considered statistically significant. The analyses were performed using SPSS statistical software version 15.0.0.1.

## Results

We received completed questionnaires from 972 (76%) of the 1,284 individuals selected for the study. Thus, the study included 504 knees with resurfaced TKA and 468 knees with non-resurfaced TKA. The response rate was similar for non-resurfaced prostheses (76%) and resurfaced prostheses (75%) (p = 0.7), but it was lower for female patients (73%) than for male patients (82%) (p = 0.001). It was also less for older patients: 88% for those less than 65 years of age and 67% for those older than 80 (p < 0.001). Response rates for each brand of prosthesis varied between 71% and 79% (Table 1, see Supplementary data). Male patients constituted 29% of the material and the mean age at the time of completing the questionnaire was 76 (SD 8) years. Table 2 gives the distribution of patient characteristics by prosthesis type and prosthesis brand.

**Table 2.** Patient characteristics by prosthesis type and prosthesis brand

	AGC	Genesis I	LCS	NexGen	Total
No. of prostheses					
Resurfaced	99	132	184	89	504
Non-resurfaced	106	134	180	48	468
All	205	266	364	137	972
No. of hospitals					
Resurfaced	16	18	9	3	40
Non-resurfaced	12	21	19	7	47
All					56
Men %					
Resurfaced	32	25	32	29	30
Non-resurfaced	32	29	24	40	29
All					29
Mean (SD) age (years) **^a^**					
Resurfaced	76 (7.2)	77 (7.1)	75 (8.1)	74 (8.1)	76 (7.7)
Non-resurfaced	76 (8.6)	78 (6.0)	76 (7.6)	74 (9.3)	76 (7.7)
All					76 (7.7)
Mean (SD) time since operation (years) **^b^**					
Resurfaced	7.2 (2.5)	9.2 (1.7)	6.5 (1.8)	5.2 (1.9)	7.1 (2.4)
Non-resurfaced	7.1 (2.4)	9.2 (1.6)	6.5 (1.8)	3.6 (0.9)	7.1 (2.5)
All					7.1 (2.4)
Charnley Category C %					
Resurfaced	57	69	61	66	63
Non-resurfaced	66	65	69	61	66
All					65
Mean (SD) preoperativ EQ-5D index score					
Resurfaced	0.48 (0.23)	0.47 (0.22)	0.44 (0.23)	0.45 (0.23)	0.46 (0.23)
Non-resurfaced	0.47 (0.20)	0.46 (0.22)	0.48 (0.23)	0.43 (0.20)	0.47 (0.22)
All					0.46 (0.22)
**^a^** Mean age when completing the questionnaire.					
**^b^** Mean time since operation when completing the questionnaire.					

**Figure 2.**
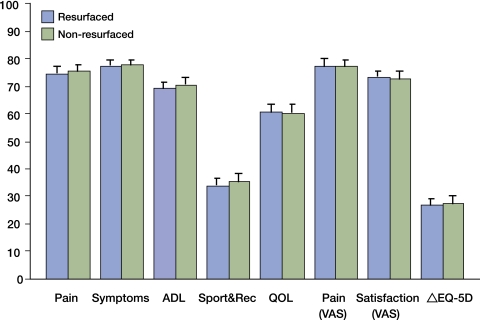
Mean outcome scores for resurfaced and non-resurfaced prostheses. The first 5 outcomes from the left represent the KOOS subscales. Adjustments were made for age, sex, preoperative EQ-5D index score (except for the outcome ΔEQ-5D), Charnley category, and prosthesis brand. Outcomes were measured on a scale from 0 to 100 units (worst to best).

**Figure 3.**
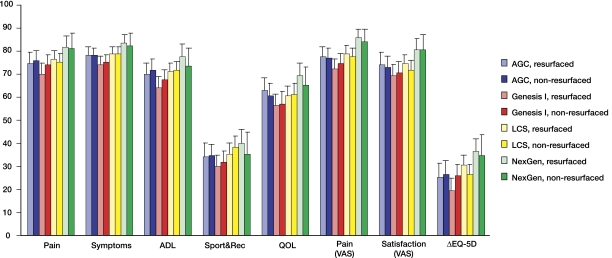
Mean outcome scores for resurfaced and non-resurfaced prostheses, for each brand of prosthesis. The first 5 outcomes from the left represent the KOOS subscales. Adjustments were made for age, sex, preoperative EQ-5D index score (except for the outcome ΔEQ-5D), and Charnley category. Outcomes were measured on a scale from 0 to 100 units (worst to best).

We observed no differences between resurfaced and non-resurfaced prostheses for any of the eight outcomes, with all p-values > 0.4 (Table 3 and Figure 2); nor did we find evidence of any differences between the 2 treatment options when analyses were performed within each brand of prosthesis (Genesis I, AGC, LCS, and NexGen) (Table 3 and Figure 3).

**Table 3.** Mean difference **^a^** in outcome between resurfaced and non-resurfaced prostheses

Prosthesis brands	Pain		Symptoms		ADL		Sport&Rec		QOL		Pain(VAS)		Satisfaction(VAS)		ΔEQ-5D	
	Δ	p	Δ	p	Δ	p	Δ	p	Δ	p	Δ	p	Δ	p	Δ	p
Total **^b^**	0.8	0.6	0.2	0.9	1.4	0.4	1.4	0.5	–0.2	0.9	-0.1	0.9	–0.7	0.7	0.4	0.9
AGC **^c^**	0.8	0.8	0.0	1.0	2.1	0.5	0.2	1.0	–1.9	0.6	–0.4	0.9	–1.2	0.7	1.4	0.8
Genesis I **^c^**	4.0	0.2	0.7	0.8	3.2	0.3	1.8	0.6	1.0	0.8	2.5	0.4	1.1	0.7	6.3	0.1
LCS **^c^**	–1.3	0.6	–0.1	1.0	0.9	0.8	2.9	0.4	1.0	0.8	–1.3	0.6	–2.5	0.4	–4.1	0.2
NexGen **^c^**	–0.6	0.9	–1.3	0.7	–4.0	0.4	–4.3	0.4	–4.3	0.4	–1.7	0.6	0.0	1.0	–1.3	0.8
**^a^** Differences (Δ) = mean scores among non-resurfaced prostheses minus mean scores among resurfaced prostheses.																
**^b^** Differences in mean are adjusted for age, sex, preoperative EQ-5D index score (except for ΔEQ-5D), Charnley category and prosthesis brand.																
**^c^** Differences in mean are adjusted for age, sex, preoperative EQ-5D index score (except for ΔEQ-5D) and Charnley category.																

Since non-resurfaced and resurfaced prostheses generally showed similar results, we did not differentiate between the two treatment groups when investigating possible effects on pain and function of prosthesis brand, sex, age, Charnley Category, time since operation, and preoperative EQ-5D index score (Table 4) (Figure 4, see Supplementary data). Genesis I and LCS did not perform statistically significantly different from the reference brand AGC, but there was a tendency of poorer results for the Genesis I for all outcomes. NexGen had a tendency of better results than AGC, but this was only statistically significant for the outcomes Symptoms (difference = 4.9, p = 0.05), Pain(VAS) (difference = 8.3, p = 0.004) and Satisfaction(VAS) (difference = 7.9, p = 0.02).

**Table 4.** Effects on mean outcome of gender, age, preoperative EQ-5D index score, time since operation, Charnley category and prosthesis brand

		Pain		Symptoms		ADL		Sport&Rec		QOL		Pain(VAS)		Satisfaction(VAS)		ΔEQ-5D	
Risk predictors	n	Δ **^a^**	p	Δ **^a^**	p	Δ **^a^**	p	Δ **^a^**	p	Δ **^a^**	p	Δ **^a^**	p	Δ **^a^**	p	Δ **^a^**	p
Sex																	
Male	284	ref		ref		ref		ref		ref		ref		ref		ref	
Female	679	–8.6	e	–6.5	e	–10.1	e	–15.5	e	–8.1	e	–6.1	e	–6.7	e	–2.9	0.2
Age (years) ^**b**^																	
<65	94	ref		ref		ref		ref		ref		ref		ref		ref	
65–70	108	7.1	0.05	4.3	0.1	3.5	0.3	1.2	0.8	2.7	0.5	4.0	0.2	0.9	0.8	–0.2	1.0
70–80	396	7.9	0.01	6.3	0.01	1.9	0.5	–1.5	0.7	3.1	0.4	7.3	0.01	1.9	0.6	–4.6	0.2
>80	365	10.9	e	10.0	e	3.5	0.3	1.8	0.6	8.4	0.02	8.5	0.004	3.0	0.4	–11.5	0.002
Preoperative EQ-5D index score **^c^**																	
<0.30	286	ref		ref		ref		ref		ref		ref		ref			
0.30–0.69	469	8.6	e	4.7	0.002	7.2	e	3.8	0.09	6.0	0.006	5.9	e	3.2	0.1		
>0.69	148	11.3	e	9.2	e	12.1	e	9.1	0.004	13.3	e	9.3	e	7.5	0.006		
Years since operation	–0.5	0.2	–0.3	0.4	–0.6	0.2	0.2	0.7	–0.1	0.8	–0.1	0.9	0.2	0.8	–0.7	0.2	
Charnley Category **^c^**																	
A	195	ref		ref		ref		ref		ref		ref		ref		ref	
B	128	–13.1	e	–9.1	e	–11.2	e	–17.8	e	–16.8	e	–13.2	e	–8.7	0.004	–11.2	0.001
C	585	–9.6	e	–6.6	e	–14.5	e	–15.3	e	–13.7	e	–10.6	e	–7.3	0.001	–5.9	0.02
Prosthesis brand																	
AGC ^**d**^	196	ref		ref		ref		ref		ref		ref		ref		ref	
Genesis I	266	–1.5	0.5	–2.9	0.2	–2.6	0.3	–3.1	0.3	–4.2	0.2	–3.2	0.2	–3.1	0.2	–0.6	0.8
LCS	364	1.2	0.6	1.2	0.5	0.9	0.7	3.4	0.2	–0.1	1.0	1.9	0.4	0.5	0.8	1.8	0.5
NexGen	137	5.4	0.08	4.9	0.05	4.0	0.2	3.9	0.3	6.5	0.07	8.3	0.004	7.9	0.02	5.9	0.1
**^a^** Differences in mean scores are adjusted for all other variables in a linear regression model.																	
**^b^** Age when completing the questionnaire.																	
**^c^** Information on preoperative EQ–5D index score was not given by 60 patients and on Charnley category by 55 patients.																	
**^d^** 9 patients not having the AGC Universal design were excluded (5 resurfaced and 4 non-resurfaced).																	
**^e^** p-value < 0.001.																	

We found that male patients performed statistically significantly better than females in all outcomes except for the ΔEQ-5D. Charnley group A performed better than both group B and C for all outcomes, while there was a positive effect of higher age except for the 2 KOOS subscales ADL and Sport&Rec and the outcome Satisfaction(VAS). Improvement as measured by ΔEQ-5D decreased, however, with increasing age (Table 4).

Exclusion of prostheses with posterior cruciate ligament sacrificing design and of constrained condylar prostheses gave only minor changes to the results above.

### Sensitivity analysis

The mean pain and discomfort score (EQ-5D) was 1.6 both for intact resurfaced and non-resurfaced prostheses (original material). For resurfaced knees, the mean score increased to 1.7 when all revised knees (n = 23) were included. The mean score was also 1.7 for non-resurfaced knees when the same number of revised knees was added (Table 5). No statistically significant difference in mean scores was observed until the number of revised knees added was more than 3 times higher for non-resurfaced knees (n > 69) than for resurfaced knees (Table 5).

**Table 5.** Sensitivity analysis of potential bias due to different revision criteria from resurfaced and non-resurfaced implants

Ratio **^a^**	Non-resurfaced			Resurfaced			Mann-Whitney
	Primary (n)	Revised (n)	Mean pain **^b^**	Primary (n)	Revised (n)	Mean pain **^b^**	p-value
– **^c^**	468	–	1.6	504	–	1.6	0.9
– **^d^**	–	145 **^e^**	2.7	–	23	2.6	0.7
1.0	468	23	1.7	504	23	1.7	1.0
1.3	468	30	1.7	504	23	1.7	0.7
1.5	468	35	1.7	504	23	1.7	0.6
2.0	468	46	1.7	504	23	1.7	0.5
2.5	468	58	1.8	504	23	1.7	0.1
3.0	468	69	1.8	504	23	1.7	0.07
3.5	468	81	1.8	504	23	1.7	0.02
**^a^** Approximate ratio between number of revised non-resurfaced and revised resurfaced implants.							
**^b^** Score from the EQ-5D question regarding pain and discomfort (1=best,3=worst)							
**^c^** Original data; patients with unrevised implants.							
**^d^** Additional data; patients with revised implants. Among the 23 revised resurfaced prostheses the most common reasons for revision were loose distal component (n=11) and pain (n=7). Among the 145 revised non-resurfaced prostheses the most common reasons for revision were loose distal component (n=38), instability (n=13), deep infection (n=14) and pain (n=72). More than one reason for revision may have been given.							
**^e^** 3 of the 148 available patients with revised non-resurfaced implants failed to answer the specific EQ-5D question.							

## Discussion

We have studied performance of TKA based on data from the NAR. Using self-reported degree of pain and function as outcome, our analyses did not show any differences between resurfaced and non-resurfaced primary total knee prostheses 2 years or more after surgery. Differences between prosthesis brands were small and did not reach the required level of minimal perceptible clinical difference relative to the reference brand, AGC.

### Strengths and limitations

The strength of our study is that use of data from a nationwide register with almost complete coverage gives us the opportunity to include several implant designs and to involve large numbers of surgeons and hospitals performing various amounts of surgery. Since this gives us information from a broad spectrum of implants, surgical techniques, surgeon experience, and procedures, the validity of the results may be more global than that from randomized controlled trials (RCTs).

Despite having several advantages, observational studies may be affected by limitations that are absent in well-designed RCTs. We have treated the most common confounding factors by using matching procedures and adjustments in the statistical model, but there may still have been variables that were not taken into account. Different criteria in the decision making by the surgeons might possibly lead to confounding. Imbalanced differences between the groups are, however, not very likely in Norway as most surgeons—for specific time periods—have used one of the two treatment options almost exclusively.

Selection bias is not very likely since the group of non-responding individuals was acceptably small and there were no statistically significantly different response rates between the most important subgroups. It is difficult to point out important factors that could have characterized the group of non-responding individuals and among those who were not eligible for inclusion, such as patients who had died.

Bias may, however, have been introduced if the study included a falsely low proportion of individuals with poor results among the non-resurfaced patients. This might be the case if non-resurfaced knees were revised more often than resurfaced knees, making such patients ineligible for inclusion in the study. This was investigated in a sensitivity analysis where information from varying numbers of revised prostheses were added to the original material. However, we observed no statistically significant difference between the treatment groups until more than 3 times as many revised non-resurfaced prostheses (n > 69) as revised resurfaced prostheses (n = 23) had been added to the material. Such a substantial difference is not supported by the results of large observational studies (Australian Orthopaedic Association National Joint Replacement Registry. Annual Report. 2008, Furnes et al. 2002), with a 30% higher rate of revision for non-resurfaced prostheses.

Since every outcome in our study, except the ΔEQ-5D, was based on the patients' perception of pain and function experienced during the previous week, we assume that the risk of recall bias was negligible.

### Explanations/mechanisms

Our results suggest that resurfacing of the patella has no effect on patients' perception of pain and function in knees with unrevised implants. This is in contrast to the existing practice in many countries where the use of a patella component is recommended, often based on higher revision rates in non-resurfaced prostheses. The higher revision rates with non-resurfaced implants may, however, be due to possible overuse of the uniquely available option of secondary resurfacing of the patella if the knee is painful, and do not necessarily indicate poorer performance regarding pain and function.

Estimated differences between prosthesis brands (as compared to the reference brand, AGC) were small and less than the stipulated minimal perceptible clinical difference of 8–10 units for KOOS subscales (Roos and Lohmander 2003) and 9–12 units for outcomes on a visual analog scale (Ehrich et al. 2000). The somewhat better results with the newer NexGen implant are interesting, but there was a limited number of hospitals and surgeons involved using NexGen. Less prosthesis wear and improved surgical techniques over the years may, however, explain some of the differences observed since mean time since operation was lower for knees with a NexGen implant. We have, however, adjusted for the time since operation in the statistical models. A possible positive effect of the search for an optimal anatomical implant during the design of the NexGen implant cannot be discounted.

Male patients performed better than female patients on all postoperative outcome measures, but we did not observe any gender differences in improvement based on pre- and postoperative EQ-5D index scores (ΔEQ-5D). While the EQ-5D index score is not necessarily strongly related to having undergone TKA, this finding is in accordance with other studies that have shown that improvement in knee scores is similar for females and males after TKA (Bourne et al. 2007), but that women perform more poorly in preoperative scores and thereby also in postoperative scores (Hawker et al. 2000, Lingard et al. 2004, Ritter et al. 2008). Some manufacturers claim that newer implants specifically designed to match a woman's knee will improve the results in female patients. This suggestion has been questioned (Ritter et al. 2008), but we could not investigate this issue as gender-specific implants were not included in this study. The observed positive effect of increasing age on outcomes that are strongly related to pain has also been seen in other studies (Elson and Brenkel 2006, Singh et al. 2008). Possible explanations such as higher expectations of younger patients, and more activity and therefore increased prosthesis wear have been suggested (Elson and Brenkel 2006). We also found that patients with unilateral knee disease and without comorbidity (Charnley category A) performed better than patients with bilateral knee disease and other systematic disease (Charnley category B and C). This supports the findings of a study based on data from the Swedish Knee Arthroplasty Register (Dunbar et al. 2004) where a modified Charnley category was found to have a significant effect on outcome questionnaires after knee arthroplasty. This emphasizes the need to take comorbidity into account when performing such outcome studies.

### Future research

Even though studies based on data from registers give a unique opportunity to discover and indicate underlying or hidden mechanisms, their limitations underscore the need for more studies. Further research performed by the use of both observational studies and clinical trials is therefore needed in order to confirm our findings, especially since our results contradict with findings in previous studies (Forster 2004, Nizard et al. 2005, Pakos et al. 2005, Parvizi et al. 2005).

### Comparison with other studies

Recent studies not included in the meta-analyses that have focused on outcomes other than revision rates, have differed in their conclusions regarding recommendation to use a patella component. 2 RCTs did not find any differences between the 2 treatment options when the Miller-Galante II system was used (Campbell et al. 2006, Burnett et al. 2007). This contrasts with the findings on Miller-Gallante II in a previous RCT study (Wood et al. 2002) included in 3 meta-analyses, where resurfacing was found to be the best choice of treatment. Another RCT investigating the Profix implant did not find evidence of any treatment option being superior to the other (Smith et al. 2008). Neither did a recent multicenter RCT, involving 1715 patients, observe any difference between paella resurfaced and non-resurfaced prostheses 2 years after surgery (Johnston et all. 2009). An open prospective multicenter study using the NexGen prosthesis concluded with a recommendation of resurfacing of the patella (Tabutin et al. 2005). In a recent study, an expected value decision analysis was used to determine the best pathway for treatment of the patella during TKA (Helmy et al. 2008). Based on data from seven RCTs, primary resurfacing of the patella was recommended.

Few studies make use of the advantages of data available from arthroplasty registers, but an 8-year-old study from the Swedish Knee Arthroplasty Register showed that resurfacing was the best choice of treatment as measured by rate of patient satisfaction with the result of the intervention, but this advantage decreased with the length of time that had passed since operation (Robertsson et al. 2000).

### Possible implications

Our study indicates a need to reconsider the widely accepted recommendation of primary resurfacing of the patella. A change in operation procedures towards less use of a patella component during primary TKA might be advisable. This will probably give the advantages of less extensive operation procedures with better preservation of the soft tissue of the patella, lower risk of revision due to infections, lower risk of patella fractures, shorter operation time, and lower cost (Furnes et al. 2002, Chalidis et al. 2007).

### Conclusion

The results of our study indicate that resurfacing of the patella has no clinical effect on pain and function after a TKA. The differences between the brands investigated were small and they were assumed to be of little importance clinically.

## Supplementary Material

Supplementary data. Table 1 and Figure 4 can be found on the www.actaorthop.org website, identification number 3061/09.
