# Supplementary material for: Does patella resurfacing really matter? Pain and function in 972 patients after primary total knee arthroplasty: An observational study from the Norwegian Arthroplasty Register
Source: Acta Orthop. 2010 Mar 31;81(1):99–107. doi: 10.3109/17453671003587069 (PMC2856212; doi:10.3109/17453671003587069)
Supplement: Supplementary file 1 [file ORT-1745-3674-81-099-s3061.doc]

**Does patella resurfacing really matter? Pain and function in 972 patients after primary total knee arthroplasty.**

**An observational study from the Norwegian Arthroplasty Register**

Stein Håkon Låstad Lygre^1^, Birgitte Espehaug^1^, Leif Ivar Havelin^1,3^, Stein Emil Vollset^2^, and Ove Furnes^1,3^

^1^The Norwegian Arthroplasty Register, Department of Orthopaedic Surgery,

Haukeland University Hospital, Bergen, Norway

^2^The Department of Public Health and Primary Health Care, University of Bergen, Norway

^3^The Department of Surgical Sciences, University of Bergen, Norway

Correspondence: stein.lygre@helse-bergen.no

Figure 4. Mean outcome scores for all prosthesis brands (resurfaced and non resurfaced pooled together). The first 5 outcomes from the left represent the KOOS subscales. Adjustments were made for age, sex, preoperative EQ-5D index score (except for ΔEQ-5D), Charnley category and time since operation. Outcomes were measured on a scale from 0 (worst) to 100 (best) units. 9 patients not having the AGC Universal design were excluded from this analyses (5 resurfaced and 4 non resurfaced).

| **Table 1 Response rates for prosthesis type and brand** | | | |
| --- | --- | --- | --- |
|  | Eligible for study | Included in study | Response rate % |
| Resurfaced |  |  |  |
| AGC | 134 | 99 | 74 |
| Genesis I | 186 | 132 | 71 |
| LCS | 238 | 184 | 77 |
| NexGen | 112 | 89 | 79 |
| All | 670 | 504 | 75 |
| Non resurfaced |  |  |  |
| AGC | 134 | 106 | 79 |
| Genesis I | 180 | 134 | 74 |
| LCS | 238 | 180 | 76 |
| NexGen | 62 | 48 | 77 |
| all | 614 | 468 | 76 |
|  |  |  |  |
| Total | 1284 | 972 | 76 |

|  |
| --- |

|  |
| --- |
|  |

|  |
| --- |
|  |

| **Figure 4.** |
| --- |
|  |
